# Idontograph Society, Pittsburgh, Pa.

**Published:** 1891-04

**Authors:** 


### PROCEEDINGS.

### Pittsburgh, Pa., Odontograph Society.

The annual meeting of the Odontograph Society was held in the parlor of Library Hall, January 22, 1891, President H. Depuy, presiding.

Dr. J. A. Calhoon read a paper entitled: "Latest Theories on Decay of the Teeth." Dr. George Shidle one entitled: "Dental Societies."

The Secretary reported forty-seven members in good standing. At this meeting thirty-two volumes were contributed to the library. The following officers were elected for the ensuing year:

President—Dr. F. S. Whitsler, Youngstown, O.

First Vice-President—Dr. W. F. Fundenberg.

Second Vice-President—Dr. W. A. Kessler.

Secretary—Dr. W. H. Fundenberg.

Treasurer—Dr. W. A. Lee.

Librarian—Dr. H. L. Reinecke.

Board of Censors—J. S. Goshorn, C. J. Phillips, L. Depuy.

Executive Committee—W. H. Fundenberg, George Shidle, C. B. Bratt.

